# Enhanced Activities of Blood Thiamine Diphosphatase and Monophosphatase in Alzheimer's Disease

**DOI:** 10.1371/journal.pone.0167273

**Published:** 2017-01-06

**Authors:** Xiaoli Pan, Shaoming Sang, Guoqiang Fei, Lirong Jin, Huimin Liu, Zhiliang Wang, Hui Wang, Chunjiu Zhong

**Affiliations:** 1 Department of Neurology, Zhongshan Hospital & Shanghai Medical College, Fudan University, Shanghai, China; 2 State Key Laboratory of Medical Neurobiology, Institutes of Brain Science & Collaborative Innovation Center for Brain Science, Fudan University, Shanghai, China; 3 Regional Health Service Center of Xujiahui, Xuhui District, Shanghai, China; Institut du cerveau et de la moelle epiniere, FRANCE

## Abstract

**Background:**

Thiamine metabolites and activities of thiamine-dependent enzymes are impaired in Alzheimer’s disease (AD).

**Objective:**

To clarify the mechanism for the reduction of thiamine diphosphate (TDP), an active form of thiamine and critical coenzyme of glucose metabolism, in AD.

**Methods:**

Forty-five AD patients clinically diagnosed and 38 age- and gender-matched control subjects without dementia were voluntarily recruited. The contents of blood TDP, thiamine monophosphate (TMP), and thiamine, as well as the activities of thiamine diphosphatase (TDPase), thiamine monophosphatase (TMPase), and thiamine pyrophosphokinase (TPK), were assayed by high performance liquid chromatography.

**Results:**

Blood TDP contents of AD patients were significantly lower than those in control subjects (79.03 ± 23.24 *vs*. 127.60 ± 22.65 nmol/L, P<0.0001). Activities of TDPase and TMPase were significantly enhanced in AD patients than those in control subjects (TDPase: 1.24 ± 0.08 *vs*. 1.00 ± 0.04, P < 0.05; TMPase: 1.22 ± 0.04 *vs*. 1.00 ± 0.06, P < 0.01). TPK activity remained unchanged in AD as compared with that in control (0.93 ± 0.04 *vs*. 1.00 ± 0.04, P > 0.05). Blood TDP levels correlated negatively with TDPase activities (r = -0.2576, P = 0.0187) and positively with TPK activities (r = 0.2426, P = 0.0271) in all participants.

**Conclusion:**

Enhanced TDPase and TMPase activities may contribute to the reduction of TDP level in AD patients. The results imply that an imbalance of phosphorylation-dephosphorylation related to thiamine and glucose metabolism may be a potential target for AD prevention and therapy.

## Introduction

Alzheimer’s disease (AD) is the most common type of dementia in adults and seriously impacts on global healthcare system along with increasingly accelerated aging process of society[1]. The exact etiology and pathogenesis of AD still remain unclear. Studies have demonstrated that cerebral glucose hypometabolism is an invariant feature in AD[2] and it occurs before the onset of disease symptoms, even for decades[3,4,5]. Further, the extent and topography of the reduction of cerebral glucose metabolism in AD patients correlate with cognitive impairment of the disease[6,7].

Thiamine diphosphate (TDP), an active form of thiamine, is a key coenzyme of three critical enzymes of glucose metabolism: α-ketoglutarate dehydrogenase complex (KGDHC), pyruvate dehydrogenase complex (PDHC), and transketolase (TK). Both TDP level[8,9,10,11] and activities of TDP-dependent enzymes[12,13,14,15] have been demonstrated to be significantly decreased in AD. Previous studies have demonstrated that thiamine deficiency can induce or aggravate AD-like pathologies, such as neuritic plaques, and hyperphosphorylation of tau, finally memory deficits[16]. Furthermore, benfotiamine supplementary, a lipid-soluble thiamine derivative, diminished AD-like pathologies and cognitive function in AD animal models[17]. However, thiamine itself supplementary has not shown a dramatic benefit in clinical trials of Alzheimer’s disease[15,18,19].

Our previous study found impaired thiamine metabolism in AD patients and altered levels of blood thiamine metabolites as an ideal diagnostic biomarker for AD[8]. The mechanism for the reduction of TDP level in AD patients remains to be clarified. In this study, we assayed the activities of three main thiamine metabolizing enzymes: thiamine diphosphatase (TDPase), thiamine monophosphatase (TMPase), and thiamine pyrophosphokinase (TPK) in AD patients and control subjects with matched age, gender, and educational background, to elucidate the underlying mechanism for the reduction of TDP level in AD.

## Materials and Methods

### Participants

The study design and procedures were approved by the Committee on Medical Ethics of Zhongshan Hospital, Fudan University. All participants or their health-givers provided their written informed consents. Briefly, 45 possible AD patients from the outpatient clinic of Department of Neurology, Zhongshan Hospital were diagnosed by neurologists specialized in dementia (Chunjiu Zhong, Guoqiang Fei, & Lirong Jin) according to DSM-IV and diagnostic guidelines for AD released by the National Institute on Aging-Alzheimer's Association workgroups[20]. Simultaneously, 38 of age, gender, and educational background matched control subjects without dementia were recruited from Zhongshan Hospital and Regional Health Service Center of Xujiahui District.

The following subjects were excluded from this study: 1) Those with thiamine and its analogue supplement in recent one month; 2) Those with the disorders of gastrointestinal tracts; 3) Those with chronic alcohol abuse; 4) Those with decreased folate and vitamin B12 levels, and thyroid function. The demographic information was showed in Table 1.

**Table 1:** Characteristics of Participants for thiamine-metabolizing enzymes assay (mean± SD).

Characteristics(mean± SD)	AD patients(n = 45)	Control subjects(n = 38)	P value
**Male/Female**	19/26	19/19	0.514
**Age (year)**	79.20±6.42 (63–89)	78.71±6.72 (63–89)	0.736
**Education (year)**	5.00±4.54 (0–16)	5.76±4.86 (0–16)	0.462
**MMSE scores**	5.62±6.28 (0–21)	27.08±2.24 (23–30)	<0.0001
**TDP (nmol/L)**	79.03±23.24(41.05–160.60)	127.60±22.65(77.76–187.50)	<0.0001
**TMP (nmol/L)**	5.33±4.44 (0–19.22)	3.81±2.91(0.28–14.33)	0.077
**Thiamine (nmol/L)**	3.62±3.87(0.53–19.35)	3.95±3.59(0.50–21.45)	0.691
**ApoE ε4 allele (%)**	24 (53.33)	3 (7.89)	<0.0001
**Fasting Blood glucose (mmol/L)**	5.43±1.52 (3.50–9.23)	6.13±1.51(4.81–13.88)	<0.05

### Clinical assessments

All subjects were assessed general cognitive function with the Mini–Mental State Examination (MMSE, Chinese version). Control subjects without dementia were identified according to MMSE scores adjusted with years of education[21]: illiterate individuals with MMSE scores >17, subjects with a primary school diploma and scores >21, and subjects with >9 years of education and scores >24. All subjects were tested for levels of blood hemoglobin, liver and kidney functions. AD patients were further received neurological examination and neuropsychological evaluation including Activity of Daily Living (ADL) and Clinical Dementia Rating (CDR) scales by enquiring patients and his/her healthcare givers. AD patients were examined by cranial MRI and/or CT scanning and assessed levels of blood folate, vitamin B12, and thyroid hormones.

### Determination of blood thiamine, TMP, and TDP

The procedure was described in details previously[17]. Briefly, 150 μl of blood samples anticoagulated with heparin were collected and immediately deproteinized with 150 μl of 7.4% perchloric acid. The 300 μl of mixture was centrifugated at 10000 rpm for 6 min at 4°C, and then the supernatant was collected and stored at −20°C until use. Thiamine and its phosphate esters were derived into thiochromes using potassium ferricyanide and separated by gradient elusion with a C18 reversed-phase analytical column (250×4.6 mm). The derivatives were measured by HPLC fluoroscopy (Agilent 1100, Santa Clara, CA) with an excitation wavelength of 367 nm and an emission wavelength of 435 nm. Blood TDP, TMP and thiamine levels were quantified using standard samples of TDP, TMP, and thiamine (Sigma-Aldrich, St. Louis, MO).

### Assay of blood TDPase, TMPase, and TPK activities

The activities of blood TDPase and TPK were determined as previously described with slight modification[11]. Briefly, 200 μl of EDTA-anticoagulated blood samples were homogenized with Tris-HCl buffer (20 mM, pH 7.4 containing 2 mM β-mercaptoethanol and 1 mM EDTA) in a Teflon-glass homogenizer. After the homogenate was centrifugated at 15,000 g for 40 min at 4°C, the supernatant was transferred for TPK activity assay, whereas the pellet was suspended in 225 μl of Tris buffer (50 mM, pH 7.4 containing 1% Triton X-100) for TDPase activity assay.

To assay TDPase activity, 360 μl of Tris buffer (50 mM, pH 7.4containing 5.5 mM MgCl_2_) was added to 100 μl of the pellet fraction. The reaction was initiated by adding 40 μl of TDP (2.5 μM, pH 7.4) at 37°C and terminated after 30 min incubation by adding 500 μl of perchloric acid (5.4%). The levels of TDP, TMP, and thiamine were then determined by HPLC. TDPase activity was calculated according to the generation of TMP content (nmol) per mg protein per minute.

To assay TPK activity, 280 μl of Tris-HC1 (180 mM, pH 7.5 containing 64 mM MgSO4) and 60 μl of ATP (500 mM, neutralized to pH 7.5) were added to 100 μl of the supernatant. The reaction was initiated by adding 100 μl of thiamine (1.0 μM), and terminated after 1 h incubation at 37°C by adding 540 μl of perchloric acid (5.4%). After the proteins were sedimented (10,000 g, 15 min), the levels of TDP, TMP, and thiamine in the supernatant were then measured by HPLC. TPK activity was represented as the generation of TDP content (nmol) per mg protein per minute.

The assay of TMPase activity was conducted according to Rao’s protocol with slight modification[22]. Briefly, 200 μl of EDTA-anticoagulated blood samples were homogenized with 1 ml of 0.32 M sucrose containing 1% Triton X-100. Then the homogenates were centrifuged at 1,000 g for 10 min at 4°C, and the supernatant was collected for TMPase activity assay.

To assay TMPase activity, 1 ml of Tris-maleate buffer (150 mM, pH 5.5, containing 4 mM MgCl_2_) was mixed with 100 μl of the supernatant and pre-incubated for 5 min at 37°C. The reaction was initiated by adding 100 μl of TMP (4.0 μM), and terminated after 1 h incubation by adding equivoluminal perchloric acid (5.0%). After the proteins were sedimented (10,000 g, 15 min), the levels of TDP, TMP, and thiamine in the supernatant were then measured by HPLC. TMPase activity was calculated according to the generation of thiamine content (nmol) per mg protein per minute.

Protein concentrations were determined with the Pierce™ BCA protein assay kit according to the manufacturer’s instructions (thermo scientific, Rockford, IL). All the enzyme activities were assayed concurrently for parallel groups of AD and control subjects, and the activity values for individual AD patients (%) were normalized to the average value of control group (100%) during the same assay. Enzyme activities were repeated twice and an average value was obtained.

### Apolipoprotein E (APOE) genotypes analysis

*APOE* allele analysis was conducted using an ABI real-time Taqman SNP genotyping assay (ABI, Life Technologies, Carlsbad, CA) according to the manufacturer’s instructions. Genomic DNA was purified from blood by using a genome extraction kit (TIANGEN, Beijing, China). Genomic DNA was used for allelic determination of SNP 112 (rs429358: GCCCCGGCCTGGTACAC) and SNP 158 (rs7412: GGCACGGCTGTCCAAGGA) of *APOE* gene. 1 μl of DNA is combined with 10 μl of 1 X final concentration of universal Taq Man PCR Master mix, 8.75 μl of sterilized water and 0.25 μl of primers for SNP 112 (rs429358) or SNP158 (rs7412). Both SNP assays were done separately on 2 plates for the same samples, the results were recorded and combined for genotypes determination.

### Statistical Analysis

SPSS software (version 18.0; SPSS Inc) was used for statistical analyses. Mann Whitney *U* test or Student t-test (two-tailed) and Chi-square test were used to compare demographic data among control subjects and AD patients. Kruskal-Wallis test and Mann Whitney *U* test were used to compare data among subgroups of control subjects and AD patients. All data are shown as the mean± SEM except for noted.

## Results

### Characterization of study participants

The demographic data are shown in Table 1 (mean ± SD). There is no significant difference in age, gender, or years of education between AD patients and control subjects (P = 0.514 for gender, P = 0.736 for age, and P = 0.462 for years of education). AD patients had significantly lower MMSE scores than control subjects (5.62 ± 6.28 *vs*. 27.08 ± 2.24, P < 0.0001). Blood TDP levels were significantly decreased in AD patients as compared with that in control subjects (79.03 ± 23.24 *vs*. 127.60 ± 22.65nmol/L, P < 0.0001) whereas TMP levels showed a trend to increase in AD patients as compared with control subjects, but the change did not reach statistical significance (5.33 ± 4.44 *vs* .3.81 ± 2.91nmol/L, P = 0.077). Neither was there significant difference in thiamine levels between the two groups (3.62 ± 3.87 *vs*. 3.95 ± 3.59 nmol/L, P > 0.05). Twenty-four of AD patients (24/45, 53.33%)were carriers of *APOE* ε4 allele (21 patients with 3/4 genotype, 3 with 4/4 genotype). The proportion of *APOE* ε4 allele carriers in AD patients was significantly higher than that in control subjects (3/38, 7.89%, 2 subjects with 2/4 genotype and 1 with 3/4 genotype; P < 0.0001). The levels of fasting plasma glucose in AD patients were significantly lower than that in control subjects (5.43 ± 1.52 *vs*. 6.13 ± 1.51mmol/L, P < 0.05).

### Increased TDPase and TMPase activities in AD patients

**Fig 1:**
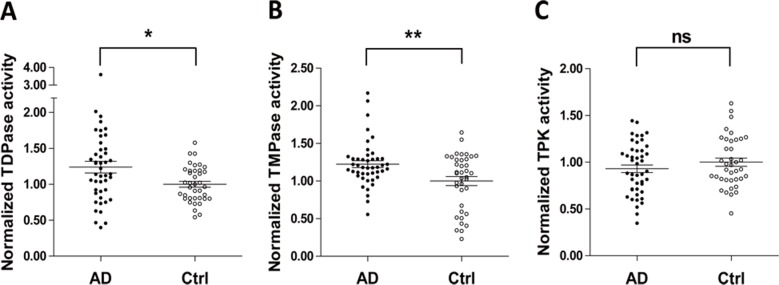
Comparison of blood TDPase, TMPase and TPK activities between AD patients and control participants. (A). Blood TDPase activity in AD (n = 45) was significantly enhanced as compared with that in control participants (n = 38, 1.24 ± 0.08 *vs*. 1.00 ± 0.04, P < 0.05;). (B). TMPase activity was markedly increased in AD patients as compared with that in control participants (1.22 ± 0.04 *vs*. 1.00 ± 0.06, P < 0.01). (C). There was no significant difference in TPK activity between AD and control subjects (0.93 ± 0.04 *vs*. 1.00 ± 0.04, P > 0.05).

TDPase, TMPase, and TPK are the main enzymes involved in thiamine metabolism. Blood TDPase and TMPaseactivities were significantly increased in AD patients as compared with those in control subjects (TDPase: 1.24 ± 0.08*vs*. 1.00 ± 0.04, P < 0.05, Fig 1A; TMPase: 1.22 ± 0.04 *vs*. 1.00 ± 0.06, P < 0.01, Fig 1B). However, blood TPK activities in AD patients were not significantly different from those in control subjects (0.93 ± 0.04 *vs*. 1.00 ± 0.04, P > 0.05, Fig 1C).

### Correlations between blood TDPase, TMPase, TPKactivities and TDP, TMP, or thiamine levels

**Fig 2:**
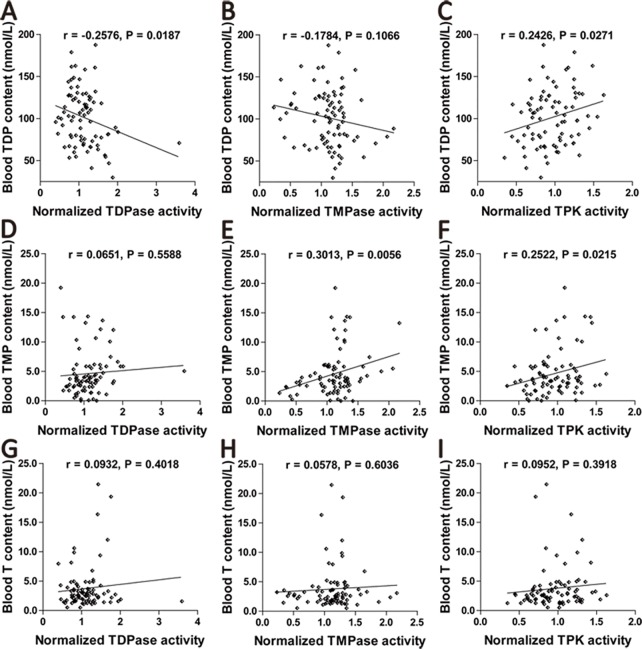
Correlations between blood TDPase, TMPase, TPK activities and TDP, TMP, thiamine levels. (A). Blood TDP levels showed a significantly negative correlation with TDPase activities in all participants (n = 83). (B). There was no significant correlation between TDP levels and TMPase activities in all participants. (C). There was a significantly positive correlation between TDP levels and TPK activities in all participants. (D). There was no significant correlation between TMP levels and TDPase activities in all participants. (E). Blood TMP contents significantly correlated with TMPase activities in all participants. (F). Blood TMP levels significantly correlated with TPK activities in all participants. (G). Blood thiamine levels did not correlate with TDPase activities in all participants (H). There was no significant correlation between thiamine levels and TMPase activities in all participants. (I). There was no significant correlation between thiamine levels and TPK activities in all participants.

The correlations between blood TDPase, TMPase, TPK activities and TDP, TMP, thiamine levels in all subjects were further analyzed (n = 83). Blood TDP levels correlated negatively with TDPase activities, whereas positively with TPK activities (TDPase: r = -0.2576, P = 0.0187; TPK: r = 0.2426, P = 0.0271, Fig 2A and 2C). There was no significant correlation between blood TDP levels and TMPase activities (r = -0.1784, P = 0.1066, Fig 2B). There was no significant correlation between blood TMP contents and TDPase activities (r = 0.0651, P = 0.5588, Fig 2D). Blood TMP levels significantly correlated with TMPase and TPK activities (TMPase: r = 0.3013, P = 0.0056; TPK: r = 0.2522, P = 0.0215, Fig 2E and 2F). Blood thiamine contents did not correlate with activities of TDPase, TMPase or TPK (TDPase: r = 0.0932, P = 0.4018; TMPase: r = 0.0578, P = 0.6036; TPK: r = 0.0952, P = 0.3918, Fig 2G–2I). Further, correlations between blood TDPase, TMPase, TPK activities and TDP, TMP, thiamine levels were analyzed respectively in AD patients and control subjects. There was a negative correlation between TDP and TDPase in AD patients (r = -0.1791) but a positive correlation in control subjects (r = 0.1059). However, both correlation did not reach a statistically difference (P = 0.2392 in AD patients, P = 0.5267 in control subjects, S1 Fig).

Further analysis was performed on the correlation between TDP and TMP contents. No significant correlation was observed in all participants or in AD patients. However, a positive correlation was observed in control subjects (S2 Fig**)**.

### Effects of fasting glucose levels on blood TDPase, TMPase, TPK activities

**Fig 3:**
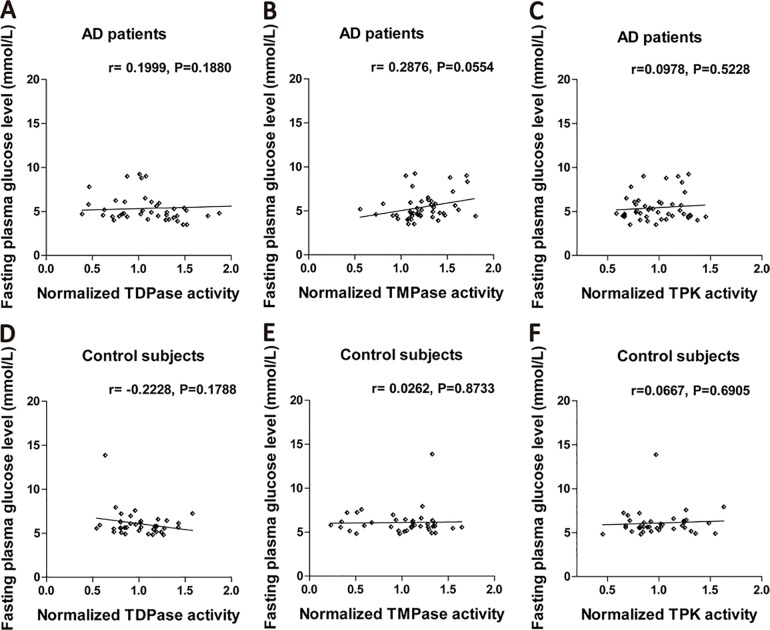
Effects of fasting plasma glucose levels on blood TDPase, TMPase and TPK activities. (A). Fasting plasma glucose levels in AD patients had no effects on the activities of TDPase (n = 45). (B). There was no significant correlation between fasting plasma glucose levels and TMPase activities in AD patients. (C). Fasting plasma glucose levels had no effects on TPK activities in AD patients. (D). Fasting plasma glucose levels in control subjects had no effects on the activities of TDPase. (E). There was no significant correlation between fasting plasma glucose levels and TMPase activities in control subjects. (F). Fasting plasma glucose levels had no effect on TPK activities in control subjects.

Fasting blood glucose levels in AD patients or control subjects had no significant correlation with the activities of TDPase (AD: r = 0.1999, P = 0.1880; control: r = -0.2228, P = 0.1788), TMPase (AD: r = 0.2876, P = 0.0554; control: r = 0.0262, P = 0.8733), and TPK (AD: r = 0.0978, P = 0.5228; control: r = 0.0667, P = 0.6905; Fig 3A–3F).

### Effects of the disease severity, age, and APOE ε4 allele on blood TDPase, TMPase, and TPK activities

To clarify the effect of disease severity on blood TDPase, TMPase, and TPK activities, AD patients were divided into different subgroups according to their MMSE (severe subgroup: MMSE<10, mild to moderate subgroup: MMSE ≥ 10) or ADL scores (severe subgroup: ADL<23, mild to moderate subgroup: ADL ≥ 23). We divided AD patients into three subgroups according to their CDR scores (severe subgroup: CDR = 3, moderate subgroup CDR = 2, mild subgroup CDR ≤ 1). No significant differences were found in TDPase activities in all subgroups based on MMSE (severe subgroup: 1.24±0.11, mild to moderate subgroup: 1.24±0.10, P = 0.86), CDR (severe subgroup: 1.27±0.12, moderate subgroup: 1.18±0.10, mild subgroup: 1.19±0.19, all P = 0.90), and ADL scores (severe subgroup: 1.33±0.14, mild-moderate subgroup: 1.15±0.08, P = 0.36, S3A–S3C Fig). Also, no significant differences were detected among all subgroups in blood TMPase or TPK activities (S3D–S3L Fig). Ages in AD patients, control subjects, or all subjects had no significant correlation with the activities of TDPase (AD: r = 0.18, P = 0.22; control: r = 0.03, P = 0.86, all subjects: r = 0.14, P = 0.22), TMPase (AD: r = 0.28, P = 0.06; control: r = 0.09, P = 0.58, all subjects: r = 0.18, P = 0.10), and TPK (AD: r = 0.11, P = 0.49; control: r = 0.13, P = 0.45, all subjects: r = 0.11, P = 0.33; S4A–S4C Fig).

Blood TDPase, TMPase, and TPK activities of *APOE* ε4 allele carriers in AD patients or control subjects were not significantly different from those in non-carriers of *APOE* ε4 allele (in AD patients, TDPase: 1.28 ± 0.13 *vs*. 1.19 ± 0.09, P = 0.60, TMPase: 1.27 ± 0.07 *vs*. 1.17 ± 0.06, P = 0.29, TPK: 0.93 ± 0.05 *vs*. 0.93±0.06, P = 0.99; in control subjects, TDPase: 0.82 ± 0.01 *vs*. 1.02 ± 0.04, P = 0.19, TMPase: 1.17 ± 0.057 *vs*.0.99 ± 0.06, P = 0.42, TPK: 1.02 ± 0.12 *vs*. 1.00 ± 0.04, P = 0.89; S4A–S4F Fig).

## Discussion

Previous studies demonstrated that thiamine deficiency can drive AD-like pathophysiological alterations. Decreased TDP levels and thiamine-dependent enzymes activities were found in AD patients but not in patients with vascular dementia, frontotemporal dementia and Parkinson’s disease[9,16]. Our previous study demonstrated that thiamine metabolism can serve as a promising biomarker for AD diagnosis with high sensitivity and specificity over 80%[8]. However, the mechanism for impaired thiamine metabolism in AD need to be clarified.

In this study, we demonstrated for the first time that blood TDPase and TMPase activities in AD patients were significantly enhanced as compared with those age- and gender-matched control subjects whereas TPK activity was not significantly changed (Fig 1). Although only TDPase and TPK, but not TMPase, activities significantly correlated with TDP levels in all subjects (Fig 2), the reduction of blood TDP levels in AD patients (Table 1) should still be attributed to the enhanced dephosphorylation due to elevated activities of TDPase and TMPase. Further, we analyzed the correlation between TDP and TMP levels respectively in AD patients and control subjects. We found that a significantly positive correlation in control subjects but not in AD patients (S2 Fig), which suggested that the balance between TDP and TMP contents was disrupted due to the enhanced TDPase and TMPase activities in AD patients. Since the three TDP-dependent enzymes, PDH, KGDH and TK, are critical for glucose metabolism, our current results strongly suggest that an impairment of phosphorylation and dephosphorylation in thiamine metabolism contributes to TDP reduction and, thus, brain glucose hypometabolism in AD.

We also investigated the association between TDPase, TMPase, and TPK activities and the disease severity evaluated by MMSE, CDR, and ADL scores in AD. Our analysis showed no significant correlations between disease severity or age and TDPase, TMPase, and TPK activities (S3 Fig and S4 Fig). Aging is a major risk factor for AD. Besides, the elderly tend to have thiamine deficiency[23,24]. However, thiamine supplementary or high thiamine dietary had a weak effect on prevention of cognitive decline with aging[25]. The finding that activities of two thiamine-metabolizing phosphatases are enhanced independent of the disease severity and age implies that the imbalance between phosphorylation and dephosphorylation of thiamine metabolism in AD is involved in pathophysiology of the disease itself rather than other illness-related changes, such as the life style and disease duration. In addition, we also excluded the possible effects of fasting plasma glucose level and APOE ε4 allele on the enzymatic activities of TDPase, TMPase, and TPK. The results showed that there were no differences in TDPase, TMPase, and TPK activities between APOE ε4-carriers and non-ε4-carriers in both control subjects and AD patients. Also, fasting plasma glucose level did not significantly correlate with blood TDPase, TMPase, and TPK activities (S4 Fig & Fig 3). Our data showed that blood TDPase activity increased by 24%, TMPase activity increased by 22% whereas TPK activity remained stable in AD patients as compared with those in control. The results differ from two previous studies[10], which showed either significantly decreased or unchanged activities of TDPase and TMPase in AD. The differences might be due to the different tissue types and the sample sizes used by the studies. The previous studies measured the enzymatic activities of TDPase and TMPase using autopsied brain tissues. The large variation in the time span from death to brain tissue collection could impact the sample quality for the measurement of enzymatic activities. Previous studies suggested that thiamine metabolizing enzymes and transporters showed different expression patterns of different organs[26,27]. Our unpublished data also showed different TPK and TDPase activities in brain, kidney, liver, blood cells and plasma of mice. Hence, sample types may be a crucial factor for enzymatic activities assay. In addition, the small sample sizes used in the previous studies could also affect the accuracy of the results.

AD pathogenesis is still unclear. Perturbed cerebral glucose metabolism is an important pathophysiological feature and precedes the onset of symptoms in AD[2,28,29,30]. Since TDP is a critical coenzyme for glucose metabolism, our study suggests that elevated TDPase and TMPase activities that lead to the reduction of TDP may contribute to brain glucose hypometabolismin AD. In addition, a previous study has demonstrated that TMPase is present only in glial enriched fractions, whereas TDPase is 20.8-fold higher in neuronal than in glial fractions[31]. It can be reasonably presumed that elevated activities of the two phosphatases originated mainly from different cell types are anon-specific consequence secondary to other AD pathophysiological alteration(s). Thus, it is possible that activities of other phosphatases are also increased in AD. Indeed, increased activities of serum ACP and ALP were observed by Vardy E and Kellete K’s studies[32]. The enhancement of non-specific phosphatase activities may be the main reason for the imbalance between phosphorylation and dephosphorylation in AD[33]. For example, increased activities of phosphatases induced dephosphorylation of glucogen synthase kinase-3 and, consequently, lead to the augmentation of its activity and tau hyperphosphorylation. Inhibiting phosphatases may be a potential target for AD prevention and treatment.

In summary, our study demonstrated that the decreased TDP level in AD was due to the enhanced TDPase and TMPase activities, which leads to elevated dephosphorylation in thiamine metabolism. TDP-dependent processes are critical for glucose metabolism, which has been demonstrated to be impaired in AD. The results indicate that an imbalance between phosphorylation and dephosphorylation in thiamine metabolism may contribute to brain glucose hypometabolism in AD and, thus, serves as a potential target for AD prevention and treatment.

## Supporting Information

S1 Dataset Original data (XLSX)

S1 Fig Correlations between blood TDPase, TMPase, TPK activities and TDP, TMP, thiamine levels respectively in AD and control subjects. (A). Blood TDP levels showed a negative correlation with TDPase activities in AD patients (n = 45) but positive correlation in control subjects (n = 38). (B). Blood TDP levels showed a negative correlation with TMPase activities in AD patients but positive correlation in control subjects. (C). There was a similar positive correlation between TDP levels and TPK activities in AD patients and control subjects. (D). Blood TMP levels showed a negative correlation with TDPase activities in AD patients but positive correlation in control subjects. (E). There was a similar positive correlation between TMP levels and TMPase activities in AD patients and control subjects. (F). There was a similar positive correlation between TMP levels and TPK activities in AD patients and control subjects. (G). There was a similar positive correlation between thiamine levels and TDPase activities in AD patients and control subjects. (H). Blood thiamine levels showed a negative correlation with TMPase activities in AD patients but positive correlation in control subjects. (I). Blood thiamine levels showed a positive correlation with TPK activities in AD patients but negative correlation in control subjects.
(TIF)

S2 Fig Correlations between blood TDP and TMP levels. (A). There was no significant correlation between TDP levels and TMP levels in all participants (n = 83). (B). Blood TDP levels showed a significantly positive correlation with TMP levels in control subjects (n = 38) but not AD patients (n = 45).
(TIF)

S3 Fig Effects of the disease severity on blood TDPase, TMPase, and TPK activities. (A). There was no significant difference in TDPase activities between two subgroups based on MMSE scores in AD patients (severe subgroup with MMSE scores < 10: 1.24 ± 0.11, n = 32; mild-moderate subgroup with MMSE scores ≥ 10: 1.24 ± 0.10, n = 13, P = 0.99). (B). No significant difference in TDPase activities were observed among three subgroups based on CDR scores (severe subgroup with CDR scores = 3: 1.27 ± 0.12, n = 29; moderate subgroup with CDR scores = 2; 1.18 ± 0.10, n = 11; mild subgroup with CDR scores ≤ 1; 1.19 ± 0.19, n = 5, P = 0.90). (C). No significant difference in TDPase activities were detected between two subgroups based on ADL scores (severe subgroup with ADL ≥ 23: 1.33 ± 0.14, n = 22; mild-moderate subgroup with ADL < 23: 1.15 ± 0.08, n = 23, P = 0.36). (D). There was no significant difference in TMPase activities between two subgroups based on MMSE scores (severe subgroup: 1.25 ± 0.06, mild-moderate subgroup: 1.17 ± 0.06; P = 0.48). (E). No significant difference in TMPase activities were detected among subgroups based on CDR scores (severe subgroup: 1.28 ± 0.06, moderate subgroup: 1.12 ± 0.07; mild subgroup: 1.16 ± 0.07; P = 0.32). (F). There was no significant difference in TMPase activities between two subgroups based on ADL scores (severe subgroup: 1.30 ± 0.07, mild-moderate subgroup: 1.15 ± 0.05; P = 0.09). (G). There was no significant difference in TPK activities between two subgroups based on MMSE scores (severe subgroup: 0.93 ± 0.05, mild-moderate subgroup: 0.92 ± 0.07; P = 0.86). (H). No significant difference in TPK activities were detected among subgroups based on CDR scores (severe subgroup: 0.96 ± 0.05, moderate subgroup: 0.92 ± 0.06, mild subgroup: 0.78 ± 0.14; P = 0.41). (I). There was no significant difference in TPK activities between two subgroups based on ADL scores (severe subgroup: 0.94±0.06, mild-moderate subgroup: 0.92 ± 0.06; P = 0.82).
(TIF)

S4 Fig Correlations between blood TDPase, TMPase, TPK activities and ages in AD patients, control subjects or all subjects. (A). There was no significant correlation between age and TDPase activities in AD patients (n = 45), control subjects (n = 38) or all participants (n = 83). (B). No significant correlation was observed between age and TMPase activities in AD patients, control subjects or all participants. (C). There was no significant correlation between age and TPK activities in AD patients, control subjects or all participants.
(TIF)

S5 Fig Effects of APOE ε4 allele on blood TDPase, TMPase and TPK activities. (A). There was no significant difference in TDPase activities between APOE ε4 allele carriers (n = 24) and non-carriers in AD patients. (B). No significant difference was observed in TMPase activities between APOE ε4 allele carriers and non-carriers in AD patients. (C). There was no significant difference in TPK activities between APOE ε4 allele carriers and non-carriers in AD patients. (D). There was no significant difference in TDPase activities between APOE ε4 allele carriers (n = 3) and non-carriers in control subjects (n = 35). (E). No significant difference was observed in TMPase activities between APOE ε4 allele carriers and non-carriers in control subjects. (F). There was no significant difference in TPK activities between APOE ε4 allele carriers and non-carriers in control subjects.
(TIF)
