# Supplementary figures and images for: Enhanced Activities of Blood Thiamine Diphosphatase and Monophosphatase in Alzheimer's Disease
Source: PLoS One. 2017 Jan 6;12(1):e0167273. doi: 10.1371/journal.pone.0167273 (PMC5218390; doi:10.1371/journal.pone.0167273)

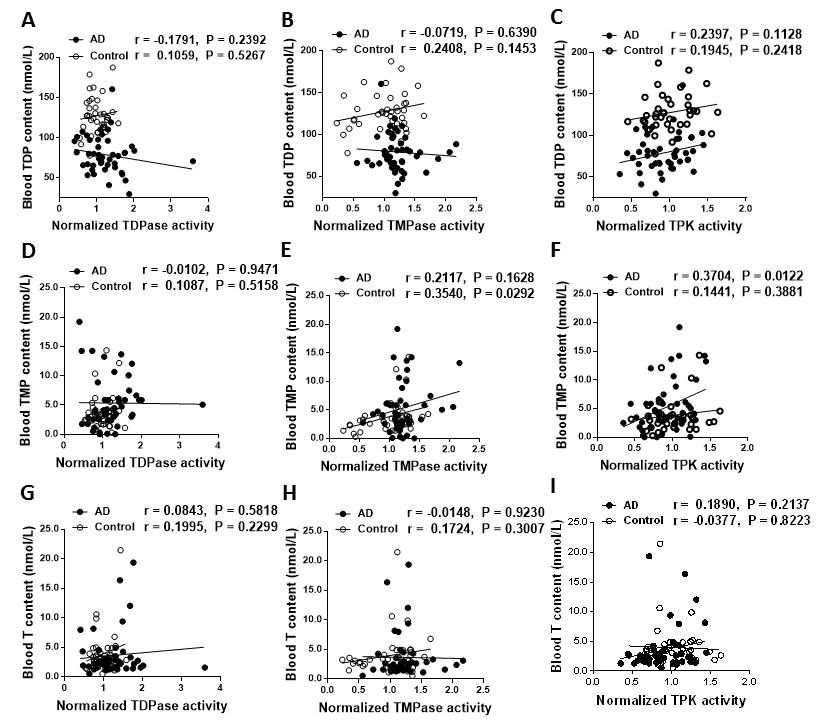

Supplement: S1 Fig — (A). Blood TDP levels showed a negative correlation with TDPase activities in AD patients (n = 45) but positive correlation in control subjects (n = 38). (B). Blood TDP levels showed a negative correlation with TMPase activities in AD patients but positive correlation in control subjects. (C). There was a similar positive correlation between TDP levels and TPK activities in AD patients and control subjects. (D). Blood TMP levels showed a negative correlation with TDPase activities in AD patients but positive correlation in control subjects. (E). There was a similar positive correlation between TMP levels and TMPase activities in AD patients and control subjects. (F). There was a similar positive correlation between TMP levels and TPK activities in AD patients and control subjects. (G). There was a similar positive correlation between thiamine levels and TDPase activities in AD patients and control subjects. (H). Blood thiamine levels showed a negative correlation with TMPase activities in AD patients but positive correlation in control subjects. (I). Blood thiamine levels showed a positive correlation with TPK activities in AD patients but negative correlation in control subjects. (TIF) [file pone.0167273.s002.tif]

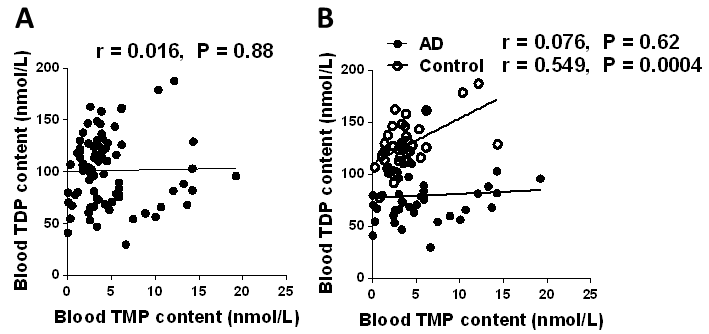

Supplement: S2 Fig — (A). There was no significant correlation between TDP levels and TMP levels in all participants (n = 83). (B). Blood TDP levels showed a significantly positive correlation with TMP levels in control subjects (n = 38) but not AD patients (n = 45). (TIF) [file pone.0167273.s003.tif]

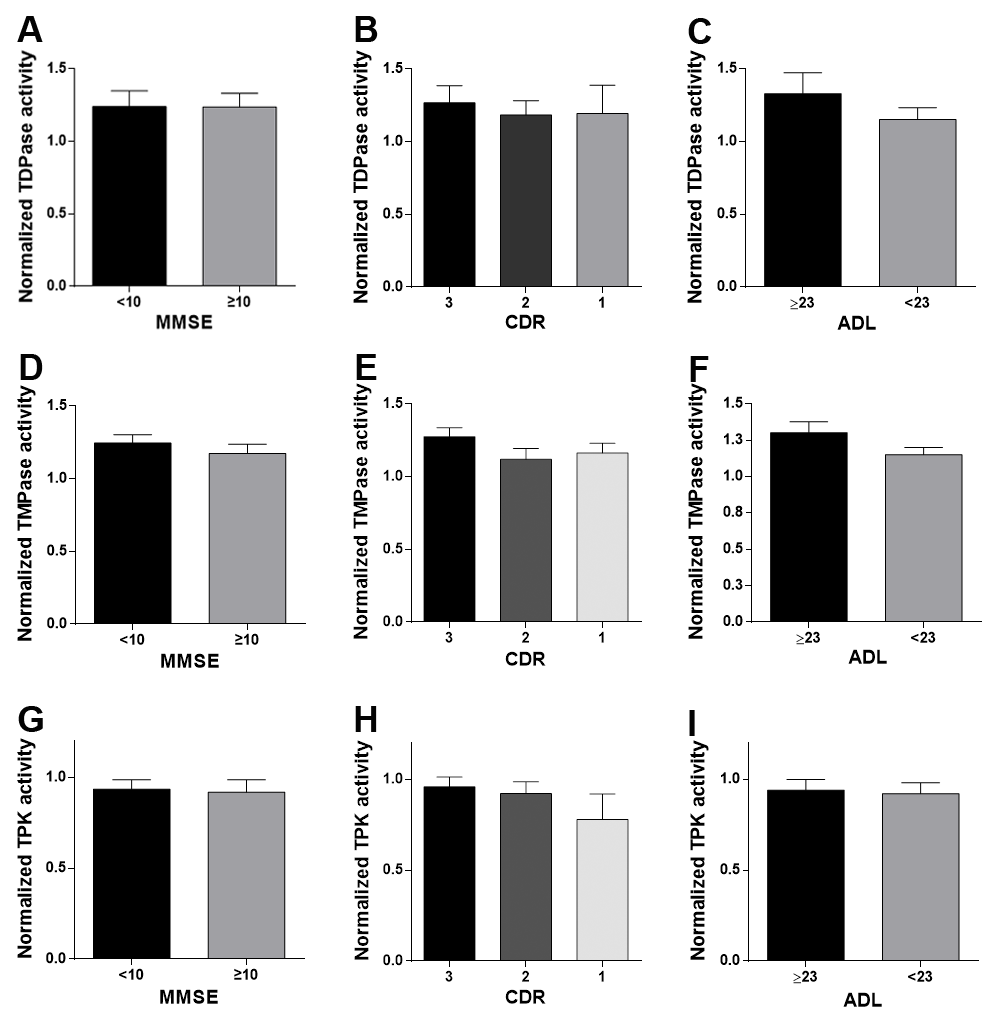

Supplement: S3 Fig — (A). There was no significant difference in TDPase activities between two subgroups based on MMSE scores in AD patients (severe subgroup with MMSE scores < 10: 1.24 ± 0.11, n = 32; mild-moderate subgroup with MMSE scores ≥ 10: 1.24 ± 0.10, n = 13, P = 0.99). (B). No significant difference in TDPase activities were observed among three subgroups based on CDR scores (severe subgroup with CDR scores = 3: 1.27 ± 0.12, n = 29; moderate subgroup with CDR scores = 2; 1.18 ± 0.10, n = 11; mild subgroup with CDR scores ≤ 1; 1.19 ± 0.19, n = 5, P = 0.90). (C). No significant difference in TDPase activities were detected between two subgroups based on ADL scores (severe subgroup with ADL ≥ 23: 1.33 ± 0.14, n = 22; mild-moderate subgroup with ADL < 23: 1.15 ± 0.08, n = 23, P = 0.36). (D). There was no significant difference in TMPase activities between two subgroups based on MMSE scores (severe subgroup: 1.25 ± 0.06, mild-moderate subgroup: 1.17 ± 0.06; P = 0.48). (E). No significant difference in TMPase activities were detected among subgroups based on CDR scores (severe subgroup: 1.28 ± 0.06, moderate subgroup: 1.12 ± 0.07; mild subgroup: 1.16 ± 0.07; P = 0.32). (F). There was no significant difference in TMPase activities between two subgroups based on ADL scores (severe subgroup: 1.30 ± 0.07, mild-moderate subgroup: 1.15 ± 0.05; P = 0.09). (G). There was no significant difference in TPK activities between two subgroups based on MMSE scores (severe subgroup: 0.93 ± 0.05, mild-moderate subgroup: 0.92 ± 0.07; P = 0.86). (H). No significant difference in TPK activities were detected among subgroups based on CDR scores (severe subgroup: 0.96 ± 0.05, moderate subgroup: 0.92 ± 0.06, mild subgroup: 0.78 ± 0.14; P = 0.41). (I). There was no significant difference in TPK activities between two subgroups based on ADL scores (severe subgroup: 0.94±0.06, mild-moderate subgroup: 0.92 ± 0.06; P = 0.82). (TIF) [file pone.0167273.s004.tif]

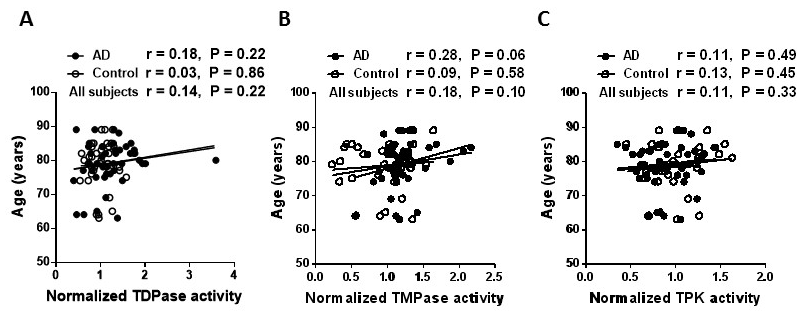

Supplement: S4 Fig — (A). There was no significant correlation between age and TDPase activities in AD patients (n = 45), control subjects (n = 38) or all participants (n = 83). (B). No significant correlation was observed between age and TMPase activities in AD patients, control subjects or all participants. (C). There was no significant correlation between age and TPK activities in AD patients, control subjects or all participants. (TIF) [file pone.0167273.s005.tif]

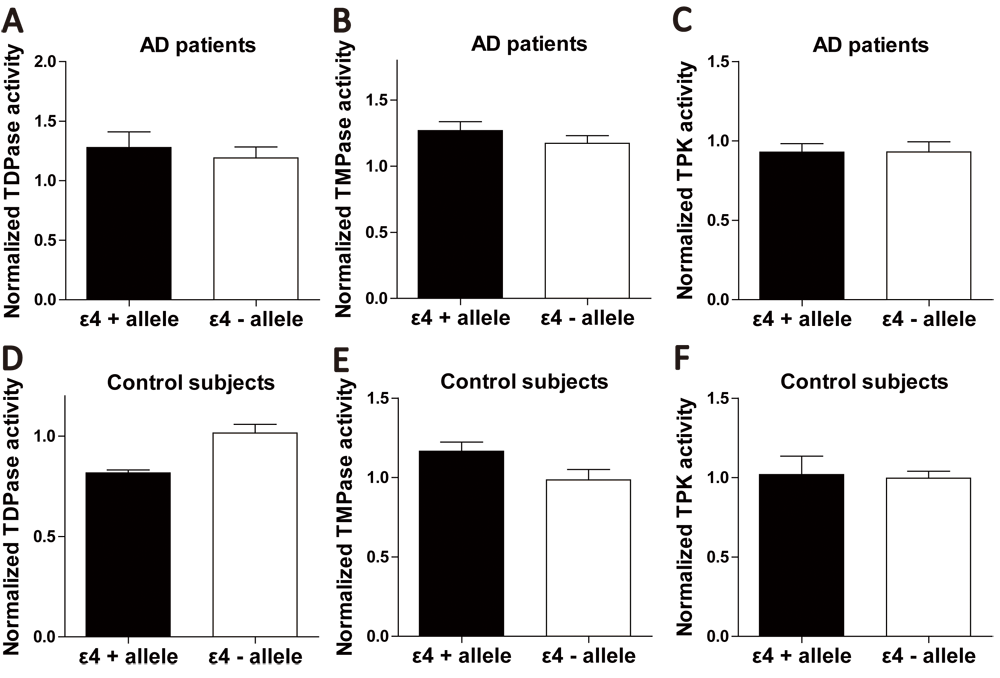

Supplement: S5 Fig — (A). There was no significant difference in TDPase activities between APOE ε4 allele carriers (n = 24) and non-carriers in AD patients. (B). No significant difference was observed in TMPase activities between APOE ε4 allele carriers and non-carriers in AD patients. (C). There was no significant difference in TPK activities between APOE ε4 allele carriers and non-carriers in AD patients. (D). There was no significant difference in TDPase activities between APOE ε4 allele carriers (n = 3) and non-carriers in control subjects (n = 35). (E). No significant difference was observed in TMPase activities between APOE ε4 allele carriers and non-carriers in control subjects. (F). There was no significant difference in TPK activities between APOE ε4 allele carriers and non-carriers in control subjects. (TIF) [file pone.0167273.s006.tif]
